# Ocular Inflammation and Oxidative Stress as a Result of Chronic Intermittent Hypoxia: A Rat Model of Sleep Apnea

**DOI:** 10.3390/antiox13070878

**Published:** 2024-07-22

**Authors:** Nina Donkor, Jennifer J. Gardner, Jessica L. Bradshaw, Rebecca L. Cunningham, Denise M. Inman

**Affiliations:** 1 Department of Pharmaceutical Sciences, University of North Texas Health Science Center, Fort Worth, TX 76107, USA; ninadonkor@my.unthsc.edu (N.D.); jennifergardner@my.unthsc.edu (J.J.G.); jessica.bradshaw@unthsc.edu (J.L.B.); rebecca.cunningham@unthsc.edu (R.L.C.); 2 North Texas Eye Research Institute, University of North Texas Health Science Center, Fort Worth, TX 76107, USA

**Keywords:** glaucoma, obstructive sleep apnea, chronic intermittent hypoxia, oxidative stress, inflammation

## Abstract

Obstructive sleep apnea (OSA) is a sleep disorder characterized by intermittent complete or partial occlusion of the airway. Despite a recognized association between OSA and glaucoma, the nature of the underlying link remains unclear. In this study, we investigated whether mild OSA induces morphological, inflammatory, and metabolic changes in the retina resembling those seen in glaucoma using a rat model of OSA known as chronic intermittent hypoxia (CIH). Rats were randomly assigned to either normoxic or CIH groups. The CIH group was exposed to periodic hypoxia during its sleep phase with oxygen reduction from 21% to 10% and reoxygenation in 6 min cycles over 8 h/day. The eyes were subsequently enucleated, and then the retinas were evaluated for retinal ganglion cell number, oxidative stress, inflammatory markers, metabolic changes, and hypoxic response modulation using immunohistochemistry, multiplex assays, and capillary electrophoresis. Statistically significant differences were observed between normoxic and CIH groups for oxidative stress and inflammation, with CIH resulting in increased HIF-1α protein levels, higher oxidative stress marker 8-OHdG, and increased TNF-α. Pyruvate dehydrogenase kinase-1 protein was significantly reduced with CIH. No significant differences were found in retinal ganglion cell number. Our findings suggest that CIH induces oxidative stress, inflammation, and upregulation of HIF-1α in the retina, akin to early-stage glaucoma.

## 1. Introduction

Glaucoma is a progressive optic neuropathy characterized by an abnormal degeneration of the retinal ganglion cells (RGCs) and their axons. It currently affects approximately 80 million individuals globally and its prevalence is anticipated to exceed 111 million by 2040 [1]. Given its asymptomatic nature, glaucoma is a leading cause of irreversible blindness with no cure [2], the latter attributed to the multifactorial nature of the disease. Conventionally, the incidence of glaucoma has been associated with impaired aqueous humor outflow and elevated intraocular pressure (IOP), leading to the use of IOP-lowering agents for the management of glaucoma over decades. However, roughly one-third of glaucoma patients have normal IOP, and some patients on IOP-lowering therapies experience unilateral blindness associated with this disease [3,4,5], indicating a need for alternative therapeutic approaches.

Recent studies have found that decreased ocular blood flow and hypoxia are crucial in glaucoma pathogenesis [6,7]. Oxygen is vital for tissue survival due to its crucial role in ATP production within mitochondria [8]. Many pathologies could impair oxygenation of ocular tissues. Among these, a higher prevalence of glaucoma has been reported in patients with obstructive sleep apnea (OSA) [9,10]. A reported 1.42-fold increased risk of developing open-angle glaucoma (OAG) in people with OSA compared to controls could be reduced following OSA surgery and/or treatment with continuous positive airway pressure [11].

OSA is a sleep disorder characterized by intermittent complete or partial occlusion of the upper airways that results in decreased airflow and oxygen desaturation. The severity of this disorder is staged using the apnea–hypopnea index (AHI), with mild (AHI: 5–15 apneic episodes per hour), moderate (AHI: 15–30 apneic episodes per hour), and severe (AHI: >30 apneic episodes per hour) divisions. Aside from glaucoma, OSA is associated with other ocular disorders such as floppy eyelid syndrome, nonarteritic ischemic optic neuropathy (NAION), and central serous retinopathy [12]. The association between glaucoma and OSA has been reported in various studies, most of which were case–control, cross-sectional, longitudinal studies or meta-analyses on the prevalence of these conditions or the correlation of OSA severity with structural and functional changes related to glaucomatous damage [13,14]. Despite considerable interest, the pathways underlying obstructive sleep apnea (OSA)-associated glaucoma remain undefined. Several hypotheses have been proposed, including a “mechanical” phenomenon, implicating nocturnal elevation of intraocular pressure (IOP) due to supine sleep posture [15], disrupted sleep hormone balance (such as the absence of a nocturnal peak in serum melatonin in OSA patients), and autonomic dysfunction [16]. Another possibility suggests ischemia or abnormal perfusion contributes to optic disc vulnerability despite normal IOP readings [5,17]. Vascular dysregulation involving alterations in vascular endothelial function, leading to vascular narrowing, reduced nitric oxide availability, and elevated endothelin-1 levels, and subsequent optic nerve ischemia and damage could also contribute [18,19,20]. Additionally, the “hypoxia/hypercarbia” theory suggests that recurrent episodes of oxygen deprivation and increased carbon dioxide levels directly harm oxygen-deprived neuronal cells, triggering systemic inflammation, oxidative stress, mitochondrial dysfunction, and ultimately ganglion cell apoptosis. 

Although there are several studies correlating the incidence of glaucoma with OSA, only a few have reported potential mechanisms involved in the phenotype observed. Fang et al. have reported the incidence of oxidative stress within the retina following severe OSA. They highlighted the role of the activation of the BDNF/TrkB signaling pathway in alleviating CIH-induced oxidative stress damage of the optic nerve and retinal ganglion cells [21]. Our current study aims to assess the metabolic, morphologic, and inflammatory changes in the retina during mild sleep apnea. Using a rat model of sleep apnea, chronic intermittent hypoxia (CIH), we hypothesized that mild sleep apnea initiates changes in the retina that resemble glaucoma-associated pathological change. Protein analysis revealed increased oxidative stress, inflammation, and hypoxia in the CIH group retina compared to normoxic controls. These findings suggest that CIH induces early-stage glaucoma-associated pathology within the retina.

## 2. Materials and Methods

### 2.1. Animals

All experiments were conducted according to the ARVO statement for the use of animals in ophthalmic and vision research guidelines and approved by the University of North Texas Health Science Center Institutional Animal Care and Use Committee (protocols 2021-0025 and 2021-0041). Tissues originated from Sprague Dawley and Long–Evans strains (Envigo, Indianapolis, IN, USA) that were being used by collaborators to investigate central mechanisms of obstructive sleep apnea. Using eyes from these animals allowed us to practice the “Reduce” directive of the National Centre for the 3Rs in animal research; the animals provided material for two studies. As such, the strain choices were driven by the central mechanism research question. A total of 32 rats provided tissues used in this study, 20 females and 12 males. The distribution of these animals across different experiments was as follows: 8 rats for capillary electrophoresis, 4 for immunohistochemistry, 12 for the thiobarbituric acid reactive substance (TBARS) assay, and 8 for the Milliplex inflammatory assay panel. For each experiment, equal numbers of rats were assigned to the normoxic and CIH groups; males and females appeared in all groups. Throughout the Results, n refers to sample size. Rats were individually housed in a temperature-controlled environment with a reverse 12:12 h light cycle (lights on at 0700 h and lights off at 1900 h). The rats were provided standard rodent chow and water ad libitum.

### 2.2. CIH Protocol

Rats were randomly assigned to either normoxia or CIH treatment groups. One week before the initiation of the CIH protocol, the home cages (clear plastic containers) of the rats were placed into Oxycycler chambers (76.2 × 50.8 × 50.8 cm, BioSpherix, Lacona, NY, USA) to acclimatize the rats to the chambers under normoxic conditions (21% oxygen). CIH was performed for 8 h starting at 2100 h during the sleep phase of the circadian cycle. The protocol consisted of oxygen reduction from 21% (room air) to 10% oxygen, then returned to 21% oxygen in 6 min cycles per hour (10 cycles/hour) over 8 h/day. For the remaining 16 h, animals were exposed to room air. This process was repeated for 10 days. Ten-day CIH tissue was used for immunohistochemistry and capillary electrophoresis. The cohort of rats exposed to the CIH protocol for 14 days yielded tissue processed for immunohistochemistry and Milliplex assay. Another cohort was exposed to the protocol for 15 days, with that retinal tissue used for lipid peroxidation analysis (TBARS). Normoxic controls were housed under similar conditions but were not exposed to oxygen modulation.

### 2.3. Sample Collection and Preparation

Animals were sacrificed within 4 to 6 h of the conclusion of CIH, between 9 am and 11 am. A subset of the rats exposed to either CIH for 14 days (*n* = 2) or normoxic (*n* = 2) conditions were transcardially flushed with 0.1 mol/L PBS and then perfused with 4% paraformaldehyde in 0.1 mol/L PBS. The eyes were enucleated and kept in 4% paraformaldehyde for 1 h, then transferred into 30% sucrose in 0.1 m PBS-0.02% sodium azide overnight, and then the cornea was removed from the globes for embedding in optimal cutting temperature (OCT) media (Fisher Scientific); globes were sectioned sagittally.

For the remaining rats, CIH (*n* = 4) and normoxia (*n* = 4), retinas were isolated and flash-frozen in liquid nitrogen. Retinal proteins were extracted with T-PER buffer containing 1x HALT protease and phosphatase inhibitors (Thermo Fisher Scientific, Waltham, MA, USA. Total protein concentration was estimated using the Bicinchoninic Acid Assay Kit (Pierce, Waltham, MA, USA) with absorbance read using a Cytation 5 (Biotek, Winooski, VT, USA) plate reader. Protein lysates were kept at −80 °C until use.

### 2.4. Immunohistochemistry

Sagittal sections of the retina (10 µm thick) were incubated in blocking buffer (5% donkey serum and 0.4% TritonX-100 in PBS) for 1 h, then with primary antibodies overnight at 4 °C. The sections were then washed three times, 10 min each, in 0.1 M phosphate-buffered saline, and following another 30 min block, secondary antibodies prepared in blocking buffer were added and incubated for 2 h at room temperature. Sections were washed again in PBS and then cover-slipped using DAPI-Fluoromount-G (Southern Biotech, Birmingham, AL, USA). Imaging of immunolabeled tissue was conducted using a Zeiss LSM 880 Airy Scan confocal microscope. Details of the antibodies used for immunohistochemistry can be found in Table 1. Proteins assessed using immunohistochemistry, including 8-Hydroxy-2-Deoxyguanosine (8-OHdG), IL-6, TNF-α, and Iba-1, were quantified by measuring the mean fluorescence intensity using ImageJ software (v. 1.54) [22]. The fluorescence intensity was normalized to the background fluorescence to ensure accurate quantification. No-primary-antibody negative control retinal sections can be found in the Supplementary Materials (Supplementary Figure S7), along with positive controls for all antibodies used for immunohistochemistry (Supplementary Figures S8–S11). Positive controls were from rat retinas subjected to ocular hypertension for four weeks using the microbead model of glaucoma as described [23]. 

### 2.5. Protein Analysis

Proteins of interest were analyzed by capillary tube electrophoresis immunoassay using the Protein Simple Jess instrument from Bio-Techne (Minneapolis, MN, USA). The proteins quantified by this method were HIF-1α, SIRTUIN-1, GLUT1, GLUT3, LDH-A, and PDK-1. Each protein was normalized to the total protein in the sample capillary according to our previous protocol [24]. Antibodies used for protein analysis by immunohistochemistry are listed in Table 1, while antibodies used for capillary electrophoresis are listed in Table 2.

### 2.6. Thiobarbituric Acid Reactive Substance (TBARS) Assay

Lipid peroxidation in retinal lysates was measured using a TBARS assay kit (Cayman Chemical, Ann Arbor, MI, USA). Standards were freshly prepared according to the manufacturer’s instructions. For each sample, 100 µL of retinal lysate was mixed with 100 µL of TCA assay reagent and 800 µL of color reagent (thiobarbituric acid (TBA) assay reagent, TBA acetic acid solution and sodium hydroxide). The mixture was vortexed thoroughly and incubated at 100 °C for 1 h. After incubation, the samples were cooled on ice for 10 min and then centrifuged at 1600× *g* at 4 °C. The supernatant was collected for analysis. Both standards and retinal lysates were plated in duplicate, and the absorbance was measured at 535 nm using a Cytation 5 plate reader (Biotek). The results are expressed as nmol/mg of protein ± SEM.

### 2.7. Milliplex Inflammation Panel

Inflammatory TNF-α levels within the rat retina were quantified using a MILLIPLEX^®^ rat cytokine/chemokine magnetic bead panel (Sigma Millipore, Cat # RECYTMAG-65 K, Temecula, CA, USA). Magnetic beads were coupled to antibodies capturing TNF-α. Retinal lysates were diluted 1:4 in assay buffer prior to conducting the assay according to the manufacturer’s instructions. The matrix solution consisted of Tissue Protein Extraction Reagent (TPER; Thermo Fisher Scientific) diluted 1:4 in assay buffer. Standards, quality controls, and retinal lysates were plated in duplicate and TNF-α cytokine levels were measured on a Luminex^®^ 200™ instrument. Quantification was performed using a five-parameter logistic curve analysis in xPONENT^®^ software version 4.3 (Luminex Corporation, Austin, TX, USA). Quality control values for TNF-α were within the range provided by the manufacturer. Values are presented as pg/mg of total protein ± SEM.

### 2.8. Statistics

Data were analyzed using GraphPad Prism v.9 (La Jolla, CA, USA). Statistical analyses were performed after evaluating normality using the Shapiro–Wilk test and then choosing the appropriate parametric or non-parametric tests. Unpaired *t*-tests were used to compare differences across groups within an outcome measure; *p* < 0.05 was considered statistically significant.

## 3. Results

### 3.1. Chronic Intermittent Hypoxia Increases Hypoxia-Inducible Factor-1α Levels

**Figure 1:**
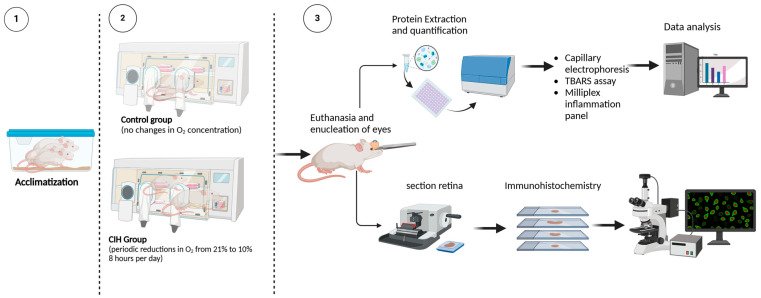
Experimental design. (1) Rats were randomly assigned to either normoxia or CIH treatment groups. Seven days before the initiation of the CIH protocol, the rats’ home cages were placed into Oxycycler chambers to acclimatize the rats to the chambers under normoxic conditions (21% oxygen). (2) CIH was performed for the CIH group over 8 h starting at 2100 h during the sleep phase of the circadian cycle. The protocol consisted of oxygen reduction from 21% (room air) to 10% oxygen, then returned to 21% oxygen in 6 min cycles per hour (10 cycles/hour) over 8 h/day. For the remaining 16 h, animals were exposed to room air. Normoxic control rats remained in the Oxycycler chambers with room air (21% oxygen) for the duration. (3) Upon completion of the CIH protocol, rats were euthanized and the retinas were analyzed for markers of inflammation and oxidative stress using immunohistochemistry, capillary electrophoresis, thiobarbituric acid reactive substance (TBARS) assay, and Milliplex immunoassay. Schematic created using BioRender.

**Figure 2:**
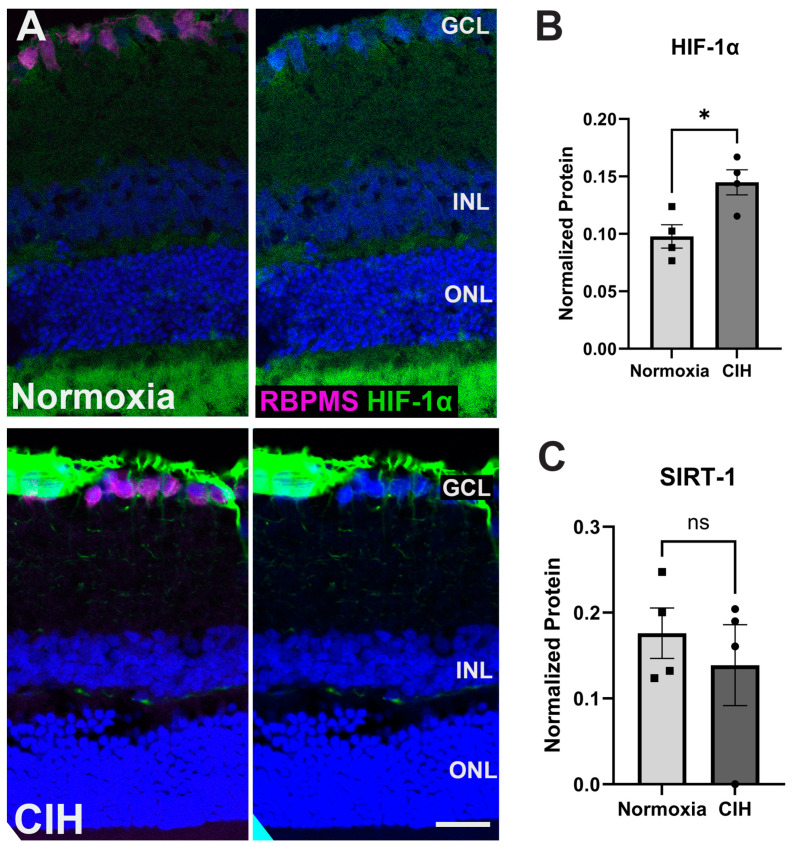
(**A**) Representative immunohistochemistry images assessing expression of HIF-1α (green) in the normoxic (upper panels) and CIH group (lower panels). Sections were also immunolabeled for retinal ganglion cells (RBPMS, magenta) and cell nuclei (DAPI, blue). Scale bar = 50 μm. *n* (sample size) = 2 rats per group. (**B**) Capillary electrophoresis (CE) assessing the protein expression of HIF-1α in the CIH group compared to normoxia showed a significant increase in HIF-1α in the CIH group (* *p* = 0.0198). (**C**) SIRTUIN-1 (SIRT-1) expression was unchanged across groups (*p* = 0.5325), as measured by capillary electrophoresis; *n* = 4 rats per group. Error bars represent mean ± SEM. GCL = ganglion cell layer; INL = inner nuclear layer; ONL = outer nuclear layer. Electropherograms of HIF-1α and SIRTUIN-1 have been included as Supplementary Materials (Supplementary Figures S1 and S2).

Figure 1 shows the experimental design for this study and subsequent data collection. To assess the effect of CIH on HIF-1α protein levels, retinal cross-sections were immunolabeled. The CIH group showed significant immunolabeling in the nerve fiber layer as compared to normoxia controls (Figure 2A). Retinal lysate was analyzed using capillary electrophoresis, resulting in a significant elevation in HIF-1α protein in the retina of rats exposed to CIH compared to the normoxic controls (*p* = 0.0198) (Figure 2B). Levels of Sirtuin-1, a regulator of Hif-1α, were somewhat reduced in the CIH group, though were not statistically different across the CIH and normoxia groups (Figure 2C). These HIF-1α increases with CIH indicate that systemic hypoxia impacted the retina.

### 3.2. Chronic Intermittent Hypoxia Increases Oxidative Stress

**Figure 3:**
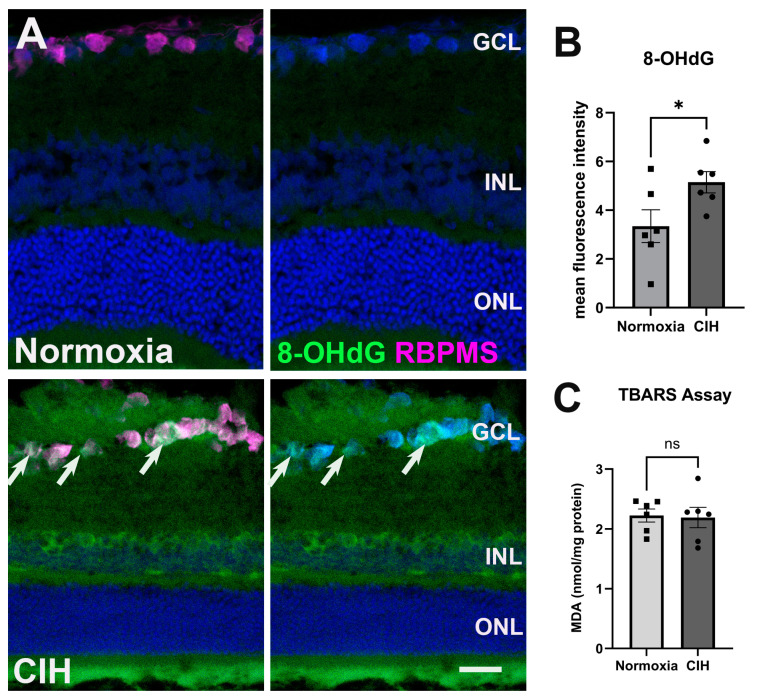
CIH induces oxidative stress. (**A**) Representative images from immunohistochemistry assessing oxidative stress using an antibody directed against 8-OHdG (green) in the normoxic (upper panels) and CIH group (lower panels). Arrows (white) point to RGCs labeled with an antibody against RBPMS (magenta) in the GCL immunolabeled with 8-OHdG. Cell nuclei labeled with DAPI (blue). Scale bar = 50 μm, *n* = 2 rats per group. (**B**) Quantified fluorescence intensity of 8-OHdG showed increased nucleic acid-associated oxidative stress damage in the CIH compared to the normoxic group (* *p* = 0.0483). (**C**) A TBARS assay quantifying lipid peroxidation in the normoxic group compared to CIH showed no difference across groups (*p* = 0.8666); *n* = 6 per group. Error bars represent mean ± SEM. GCL = ganglion cell layer; INL = inner nuclear layer; ONL = outer nuclear layer.

CIH stimulates the production of reactive oxygen species, ultimately inducing oxidative stress in the CNS [25], so we evaluated whether CIH could lead to similar alterations in the retina. Cellular membranes, due to their high concentration of polyunsaturated fatty acids (PUFAs), are especially susceptible to ROS damage [26]. Retinal cross-sections were assessed for oxidative stress-associated damage of nucleic acid using an antibody against 8-Hydroxy-2-Deoxyguanosine (8-OHdG) (Figure 3A). Significant labeling was observed in the ganglion cell and inner nuclear layers in the retina of rats exposed to CIH compared to the normoxic controls, with a statistically significant increase in 8-OHdG fluorescence intensity in the CIH group compared to normoxic control (Figure 3B; *p* = 0.0483). We further quantified lipid peroxidation in retinal lysates using the thiobarbituric acid reactive substance (TBARS) assay, showing that the CIH group had no different lipid peroxidation than the normoxia group (Figure 3C; *p* = 0.8666). This suggests that the oxidative stress engendered by CIH showed preference for nucleic acid over lipid damage.

### 3.3. Chronic Intermittent Hypoxia Induces Inflammation

**Figure 4:**
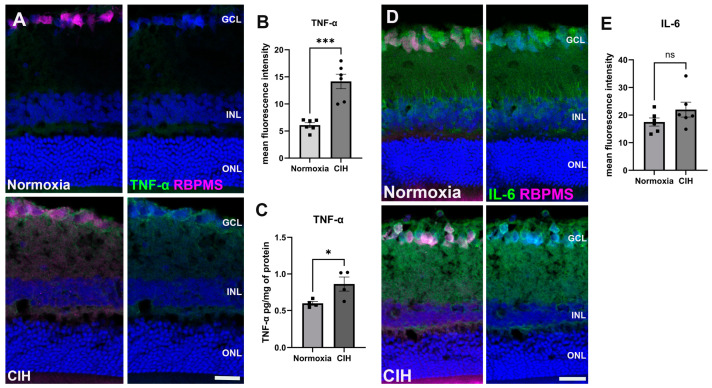
CIH induces retinal inflammation. (**A**) Representative immunohistochemistry images showing the expression of the cytokine TNF-α (green) in the normoxic (upper panels) and CIH groups (lower panels). Retinas were colabeled with RBPMS, specific for retinal ganglion cells (magenta), and also stained with DAPI for cell nuclei (blue). (**B**) Quantification of fluorescence intensity showed increased expression of the cytokine TNF-α in the CIH group compared to normoxia control (*** *p* = 0.0002; *n* = 2 rats per group. (**C**) ELISA showed a significant increase in TNF-α in the CIH group over normoxia (* *p* = 0.0379, *n* = 4 rats per group). (**D**) IHC images of IL-6 (green) in the normoxic (upper panels) and CIH group (lower panels). RBPMS (magenta) and DAPI (blue). (**E**) Quantifying IL-6 fluorescence intensity showed comparable expression of IL-6 in both normoxia and CIH groups. Error bars represent mean ± SEM. GCL = ganglion cell layer; INL = inner nuclear layer; ONL = outer nuclear layer. Scale bar = 50 μm, *n* = 2 rats per group.

CIH is known to trigger systemic inflammation [27]. We assessed inflammation in retinal sections using immunohistochemistry, labeling for tumor necrosis factor-α (TNF-α) (Figure 4A). Upon quantification of the immunolabel, we found that TNF-α was significantly increased in the CIH group compared to the control (*p* = 0.0002) (Figure 4B). We also assessed interleukin-6 (IL-6) using IHC (Figure 4C). There were no significant differences in IL-6 protein in the normoxia versus CIH groups (Figure 4D).

### 3.4. Chronic Intermittent Hypoxia Induces Microglia Activation

**Figure 5:**
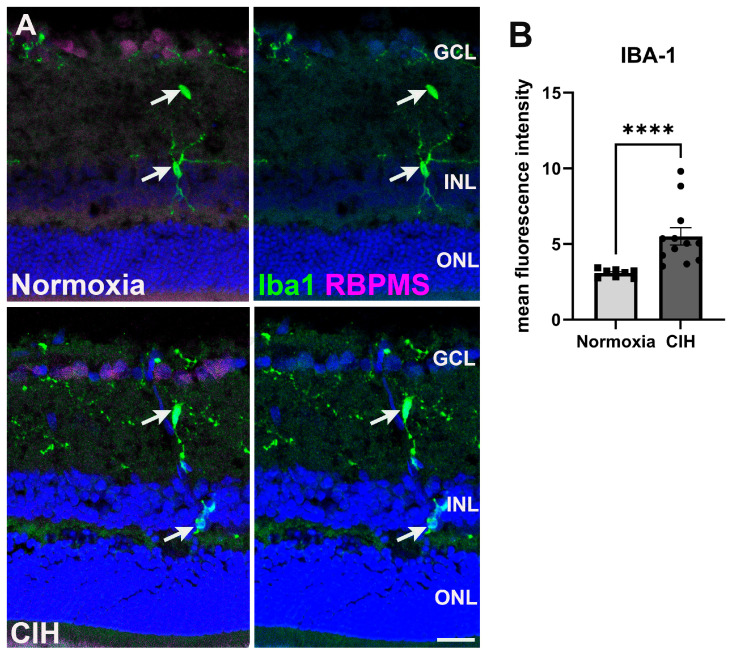
(**A**) Microglia immunolabeled using Iba-1 (green) in the normoxic (upper panels) and CIH group (lower panels). Retinal ganglion cells (magenta) and cell nuclei (DAPI, blue) are labeled for context. Arrows (white) point to Iba1-positive microglia somata. (**B**) Quantification of fluorescence intensity showed elevated levels of Iba-1 in the CIH group compared to the control (**** *p* = 0.0001). Error bars represent mean ± SEM. GCL = ganglion cell layer; INL = inner nuclear layer; ONL = outer nuclear layer. Scale bar = 50 μm, *n* = 2 rats per group.

Microglia are often activated in response to ocular insults [28]. Such activation drives inflammation that contributes to the loss of retinal ganglion cells as observed in glaucoma [29]. We labeled microglia using an antibody against ionized calcium binding adaptor molecule 1 (Iba-1) (Figure 5A). Quantifying the fluorescence intensity within the CIH and normoxic groups, we observed that Iba-1 (Figure 5B) was significantly increased in the CIH group compared to normoxia (*p* < 0.0001), indicating that CIH induces inflammation within the retina. We did investigate whether CIH promoted T-cell infiltration into the retina by immunolabeling for CD3; however, we found no evidence of T-cell infiltration into normoxic or CIH retinas from immunolabeling for CD3.

### 3.5. Effect of Chronic Intermittent Hypoxia on RGC Count

**Figure 6:**
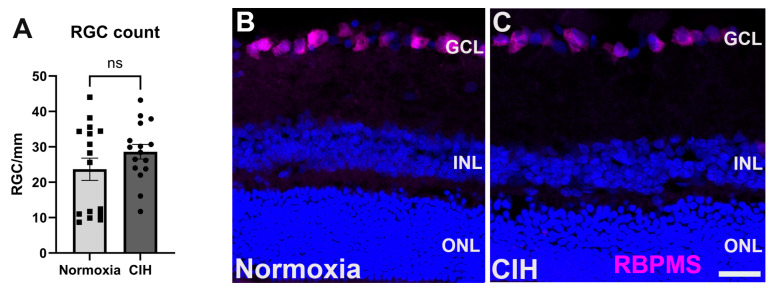
(**A**) Quantification of RGC somata in the normoxic and CIH groups (*p* = 0.3414) shows that this degree of hypoxia is not sufficient to lead to RGC apoptosis. RGCs were counted in retinal sections, with each point representing a separate section; numbers are expressed as cells per mm of GCL length. (**B**) Representative immunolabeling from normoxia and (**C**) CIH retina with RGCs immunolabeled with RBPMS (magenta) and DAPI for cell nuclei (blue). Scale bar = 20 μm.

In glaucoma, retinal ganglion cells (RGCs) undergo apoptosis as a result of intraocular pressure-associated stresses and pathological change, including hypoxia [23,30]. We assessed the impact of CIH on retinal ganglion cell survival by counting RCG somas in normoxia versus CIH retinas. We observed that this degree of CIH exposure was not sufficient to lead to RGC apoptosis; the RGC number was no different between the normoxia and CIH groups (Figure 6A). Representative immunolabeling from sectioned retinas shows RGCs in the ganglion cell layer of both normoxia and CIH groups (Figure 6B).

### 3.6. Effect of Chronic Intermittent Hypoxia on Metabolism

**Figure 7:**
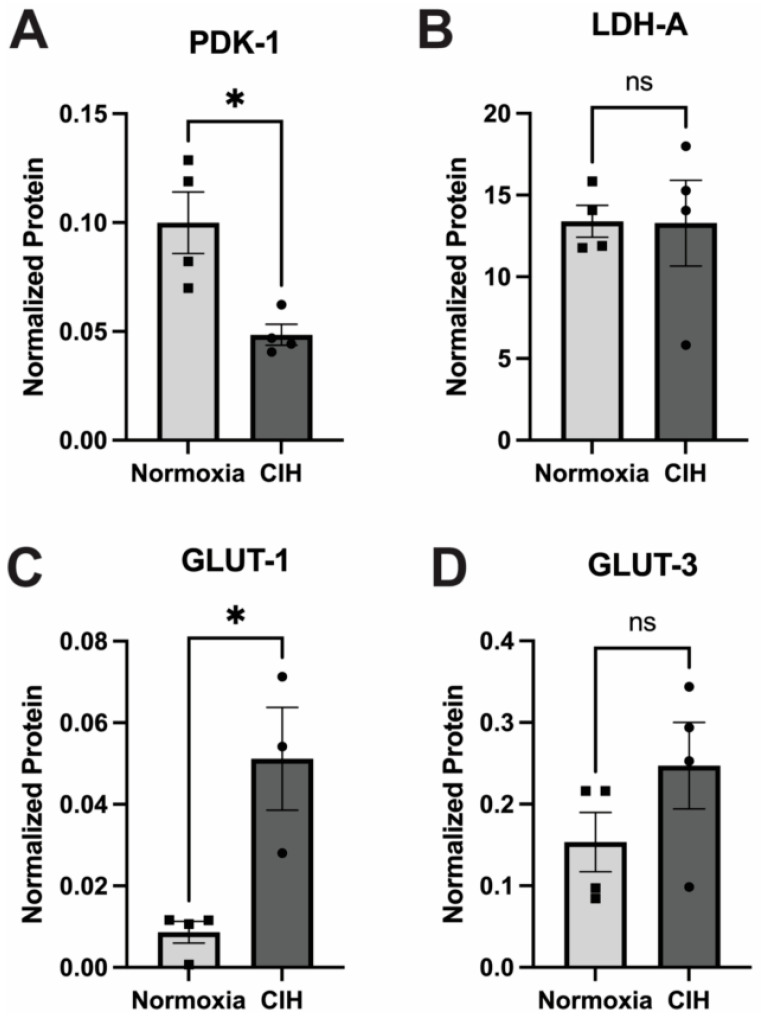
Effect of CIH on retinal metabolic enzymes and transporters as measured by capillary electrophoresis. (**A**) PDK-1 protein was significantly reduced in the CIH group retina compared to the control (* *p* = 0.03). (**B**) LDH-A protein levels were not affected by exposure to CIH (*p* = 0.9692). (**C**) The expression of GLUT-1 was significantly increased in CIH compared to the normoxia control group (* *p* = 0.0118). (**D**) GLUT-3 protein was not different across CIH and normoxia control groups (*p* = 0.2007). Error bars represent mean ± SEM; GCL = ganglion cell layer; INL = inner nuclear layer; ONL = outer nuclear layer; *n* = 4 rats per group. Electropherograms of LDH-A, PDK-1, GLUT1, and GLUT 3 have been included as Supplementary Materials (Supplementary Figures S3–S6).

The impact of CIH on cellular glucose metabolism was assessed by quantifying the expression of the enzymes pyruvate dehydrogenase kinase-1 (PDK-1) and lactate dehydrogenase-A (LDH-A) using capillary electrophoresis. PDK1 and LDHA serve pivotal roles in cellular metabolism by modulating the fate of pyruvate derived from glycolysis [31]. PDK1 phosphorylates and deactivates pyruvate dehydrogenase, inhibiting the conversion of pyruvate to acetyl-CoA, thereby favoring lactate production over entry into the TCA cycle during conditions of low oxygen availability [24]. Expression of PDK-1 in CIH retinal lysates was significantly lower (*p* = 0.03) than the normoxic control (Figure 7A). LDHA facilitates the conversion of pyruvate to lactate, aiding in anaerobic energy production and NAD+ regeneration. No significant differences were observed in the expression of LDH-A between the CIH-exposed group and normoxic controls (Figure 7B). There was a significant increase in the expression of the insulin-independent, glial cell-enriched glucose transporter GLUT 1 (*p* = 0.0118) in the CIH group (Figure 7C), though there was no change in the insulin-independent, neuronal-enriched GLUT 3 (Figure 7D) protein across the CIH and normoxia control retinas.

## 4. Discussion

Hypoxia–reoxygenation stress poses a significant threat to cellular health as evidenced by chronic diseases, including obstructive sleep apnea. CIH is an important factor contributing to various retina and optic nerve disorders associated with obstructive sleep apnea [21]. The mechanisms involved in the development of glaucoma in CIH patients are likely associated with the mechanisms contributing to systemic pathology identified in human studies, including oxidative stress and inflammation. In this study, we modeled mild sleep apnea in rats and evaluated the impact of this pathology on the retina. We sought to gain insights into the potential pathways linking CIH to retinal damage, particularly focusing on aspects relevant to glaucoma development.

Elevated levels of HIF-1α protein have been reported in the retina and ON from glaucoma patients and animal models [32]. This transcription factor is ubiquitously expressed in various cell types [33]. Under normoxic conditions, HIF-1α is constantly hydroxylated at specific proline residues by a family of enzymes called prolyl hydroxylases (PHDs) and then degraded via the ubiquitin–proteosome pathway. HIF-1α accumulates under conditions of hypoxia or pseudohypoxia [34], including in glaucoma [23,24], initiating a wide range of physiological responses, including erythropoiesis, glycolysis, and angiogenesis. By controlling the expression of genes responsible for glycolysis, HIF-1 promotes the adaptation of cells to oxygen shortage [35]. These reactions are intended to swiftly reverse the consequences of an oxygen shortage [36]. As anticipated, CIH induced an increase in the levels of HIF-1α in the retina, particularly in the nerve fiber layer and the basal processes of Müller glia. This pattern of expression has been observed after ocular hypertension in multiple models of glaucoma [29,30]. The periodic cycles of hypoxia and reoxygenation during CIH may have been sufficient to suppress the activity of prolyl hydroxylases. We quantified the expression of sirtuin-1 (SIRT-1), an NAD+-dependent deacetylase that catalyzes the deacetylation of acetyl-lysine residues on proteins such as HIF-1α. SIRT-1 activity decreases levels of HIF-1α in circulation [37,38]. We observed a reduction in the levels of SIRT-1 in the CIH group compared to the control. Though not a statistically significant decrease, reduced SIRT-1 is consistent with the HIF-1α elevation observed in the CIH group.

Previous studies in our lab have shown that the stabilization of HIF-1α using the prolyl hydroxylase inhibitor Roxadustat increased lactate metabolism [24]. We observed that mice exposed to CIH had comparable levels of LDH-A regardless of Hif-1α stabilization. Though this one outcome measure is not definitive for lactate production (it is but one isoform), we also show a significant decrease in PDK-1 in the CIH group, which implies lowered promotion of lactate production. In another study where we assessed the impact of HIF-1α stabilization on mitochondrial homeostasis and oxidative stress in a chronic model of glaucoma, we observed that the expression of LDH-A was unchanged regardless of HIF-1α stabilization [39]. This raises the prospect that the duration of HIF1α stabilization has a varied impact on lactate metabolism. HIF-1α also regulates the expression of glucose transporters by promoting their expression to support the use of glycolysis during low-oxygen conditions. We observed an increase in GLUT-1, which is found on retinal vasculature and glial cells, after CIH, but no change in GLUT-3, which is neural-specific. Our previous work documented that Hif-1α activation after ocular hypertension led to significant increases in GLUT-1 in retinas [39]. Our finding is corroborated by others who also observed no change in GLUT-3 protein expression but elevated levels of GLUT-1 in hypoxia [40]. GLUT3 is expressed specifically on neurons [41], so its lack of alteration in this model of CIH suggests that retinal neurons were able to meet their metabolic needs either without an increase in glycolysis or perhaps through upregulation of other glucose transporters such as the insulin-dependent GLUT-4. Glia are very sensitive to modulation of oxygen, and their response is among the earliest events in retinal [29] and systemic hypoxia [42].

Oxidative stress resulting from CIH is considered the hallmark of obstructive sleep apnea [43]. Oxidative stress has also been strongly linked to mitochondrial abnormalities and reduced total antioxidant status in primary open-angle glaucoma [44]. Oxidative stress and the resultant reactive oxygen species (ROS) play a significant role in the pathophysiology of glaucoma, primarily by causing damage to glial cells, disrupting autophagy processes, activating NF-κB signaling pathways, inducing nitrite stress, and altering ocular hemodynamics [45,46]. NADPH oxidase contributes to ROS in glaucoma [47], and its inhibition has been shown to alleviate retinal inflammation [48]. These processes collectively contribute to inflammation and the subsequent damage to the optic nerve or death of retinal ganglion cells (RGCs). We assessed oxidative stress in the retina using 8-OHdG and lipid peroxidation. We observed significant 8-OHdG immunolabeling in the ganglion cell layer of retina sections from the CIH group, supporting our hypothesis that CIH induces early glaucoma-like features. Significantly increased levels of 8-OHdG have been observed in POAG cohorts compared to controls; in fact, there is a positive correlation between increased levels of 8-OHdG and the risk of POAG (>4-fold) [49]. CIH promotes learning and memory impairment and insulin resistance through increased ROS production, which triggers the activation of NF-κB and subsequently activates the NLRP3 inflammasome in microglial cells [50,51]. Again, through activation of NF-κB, reactive oxygen species increase the transcription of HIF-1α [52].

The retina is composed of various lipids including phospholipids, long-chain polyunsaturated fatty acids (LCPUFAs), saturated fatty acids (SFAs), and monounsaturated fatty acids (MUFAs). LCPUFAs are essential components of ocular structures and functions, and they are part of the processes that cause or resolve inflammation by oxidized lipids [53]. This feature makes the retina highly susceptible to lipid peroxidation. Decreased total antioxidant status, increased malondialdehyde (MDA), and increased H_2_O_2_ are independent risk factors for glaucoma [54,55,56]. We did not observe significant increases in MDA, a product of lipid peroxidation, in the CIH group. Lipid peroxidation associated with OSA is dependent on the duration and/or severity of CIH [57].

Oxidative stress results in pathologic changes, including inflammation [58]. We assessed inflammation using immunohistochemistry and observed significantly elevated levels of TNF-α across the retina in the CIH group. Systemic inflammation, including increased TNF-α, has been identified as a consequence of CIH [59]. Our observation of increased TNF-α levels has also been reported in patients with OSA [60]. TNF-α is believed to facilitate cell death by binding to TNFR1, subsequently activating both the mitogen-activated protein kinase (MAPK) pathway and the canonical nuclear factor-κB (NF-κB) pathway. These activations collectively result in the transcriptional upregulation of pro-inflammatory genes, which are fundamental to the underlying inflammatory pathology [61]. Increased levels of both TNFR and TNF-α confirmed a pro-inflammatory state within tissue exposed to CIH [62].

Multiple types of glial cells are activated in glaucoma [63]. Microglia, for instance, orchestrate the progression of neuroinflammation in the CNS through pro-inflammatory cytokines [64]. Microglia possess Toll-like receptors (TLRs) on their membranes that promote inflammation when activated through the release of pro-inflammatory cytokines as observed in glaucoma and other neurodegenerative diseases such as Alzheimer’s disease [65]. We quantified microglial activation in the retina and our finding suggests that microglial activation occurred in the CIH group, likely contributing to the increase in TNF-α observed. However, specific analyses of morphology and activation markers are warranted in future studies.

The eye is an immune-privileged organ; however, in mouse glaucoma models and humans with glaucoma, T-cell infiltration is observed [66,67]. T-cell responses have been considered essential in the development of progressive glaucomatous neurodegeneration [68]. Many studies have demonstrated that CIH induces endothelial dysfunction [69,70]. We assessed retinal sections for T-cell infiltration following CIH exposure. However, we did not observe T cells in either group.

RGC death is among the key features of glaucoma pathogenesis [71], contributing to the loss of peripheral vision observed in glaucoma patients [72]. We quantified and compared RGC somas across our groups and observed no difference. This is likely a result of our use of a mild CIH model. A model of CIH mimicking moderate or severe OSA might result in the death of RGCs, as this has been previously demonstrated [21]. Regardless, investigating other pathways that may be initiated prior to cell death that are impacted by hypoxia and implicated in glaucoma (e.g., autophagy, mitophagy, senescence) is an avenue for future studies. This study did not investigate sex differences because the limited male and female sample size did not allow for sufficient power to detect statistical differences between sexes. Future research will focus on assessing potential sex differences in the observed early-glaucoma-like changes in the retina following mild OSA.

Limitations of this study include the evaluation of pathological changes for mild OSA alone, and in a relatively short time frame (10–15 days) of exposure to CIH. For most patients, OSA is a chronic condition that may or may not treated, particularly for those with a mild apnea–hypopnea index (15 or fewer apneic episodes per hour). This investigation suggests that mild OSA modestly elevates HIF-1α, oxidative stress, and inflammation, yet it remains to be determined if these changes contribute in meaningful ways to ocular conditions such as glaucoma. Examination of models for moderate and perhaps severe OSA, with longer bouts of CIH (greater than 15 days), could reveal a more definitive link.

## 5. Conclusions

In summary, our data provide evidence that mild CIH increases inflammation, oxidative stress, and HIF-1α levels within the retina. Specifically, we observed elevated levels of inflammatory markers such as TNF-α, increased oxidative stress indicated by 8-OHdG levels, and upregulation of HIF-1α and its downstream target, GLUT1. These may be the major pathways through which obstructive sleep apnea is linked to the pathogenesis of glaucoma.

## Figures and Tables

**Table 1:** Antibodies used for immunohistochemistry.

Antigen	Species	Manufacturer	Catalog Number	Dilution
Hif-1α	Rabbit	Novus	NB100-134	1:500
Iba-1	Rabbit	Wako/Sigma	019-19741	1:250
RBPMS	Rabbit	Genetex	GTX 118619	1:250
	Mouse	Novus	OTI3B7	1:250
TNF-α	Mouse	Abcam	Ab1793	1:50
8-OHdG	Mouse	QED Bioscience	12501	1:100
IL-6	Rabbit	Invitrogen	PA5-120041	1:100

**Table 2:** Antibodies used for capillary electrophoresis.

Antigen	Species	Manufacturer	Catalog Number	Dilution
Hif-1α	Rabbit	Novus	NB100-134	1:25
PDK1	Rabbit	Cell Signaling	C47H1	1:100
LDH-A	Rabbit	Novus	NBP1-48336	1:100
SIRT-1	Rabbit	Novus	MBP2-27205	1:100
GLUT 1	Rabbit	Novus	NB110-39113	1:50
GLUT 3	Mouse	R&D Systems	MAB1415	1:25

## Data Availability

The original contributions presented in this study are included in the article. Further inquiries can be directed to the corresponding author.

## Supplementary Materials

The following supporting information can be downloaded at: https://www.mdpi.com/article/10.3390/antiox13070878/s1, Supplementary figures of electropherograms for capillary electrophoresis (Supplementary Figures S1–S6), as well as negative controls (Supplementary Figure S7) and positive controls (Supplementary Figures S8–S11) for immunohistochemistry
