# Supplementary material for: Ocular Inflammation and Oxidative Stress as a Result of Chronic Intermittent Hypoxia: A Rat Model of Sleep Apnea
Source: Antioxidants (Basel). 2024 Jul 22;13(7):878. doi: 10.3390/antiox13070878 (PMC11273423; doi:10.3390/antiox13070878)

Supplementary Data

Electropherograms from capillary electrophoresis

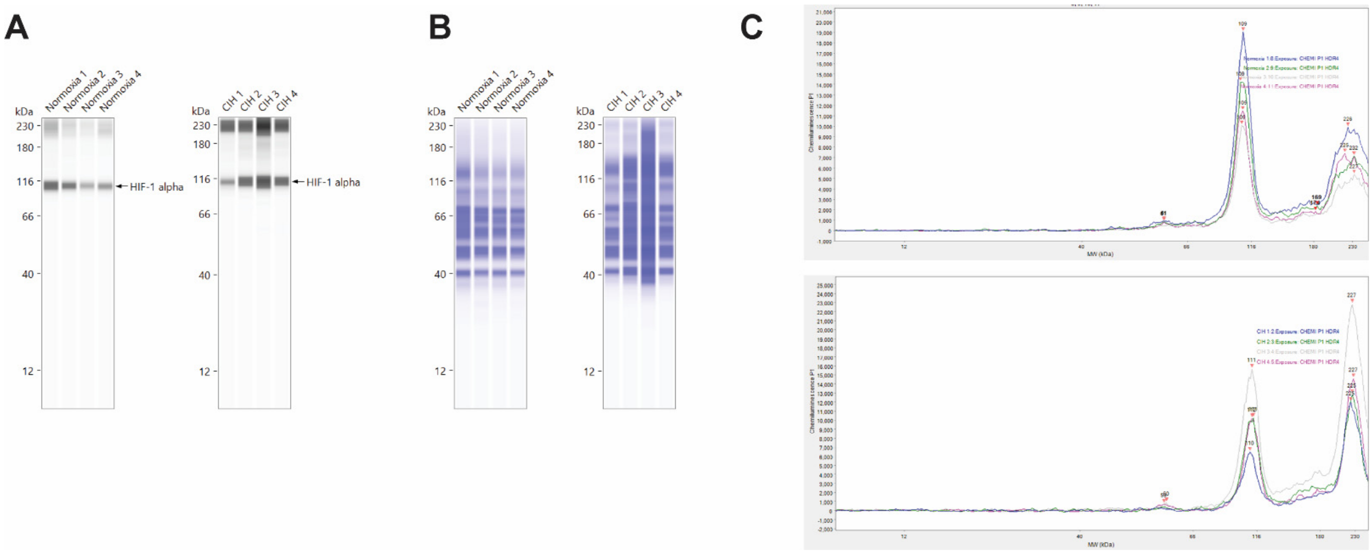

Supplementary Figure S1: Electropherograms from capillary electrophoresis assessing HIF-1α protein expression. A) Lane view of HIF-1α protein expression B) Total protein per sample C) Graphical view

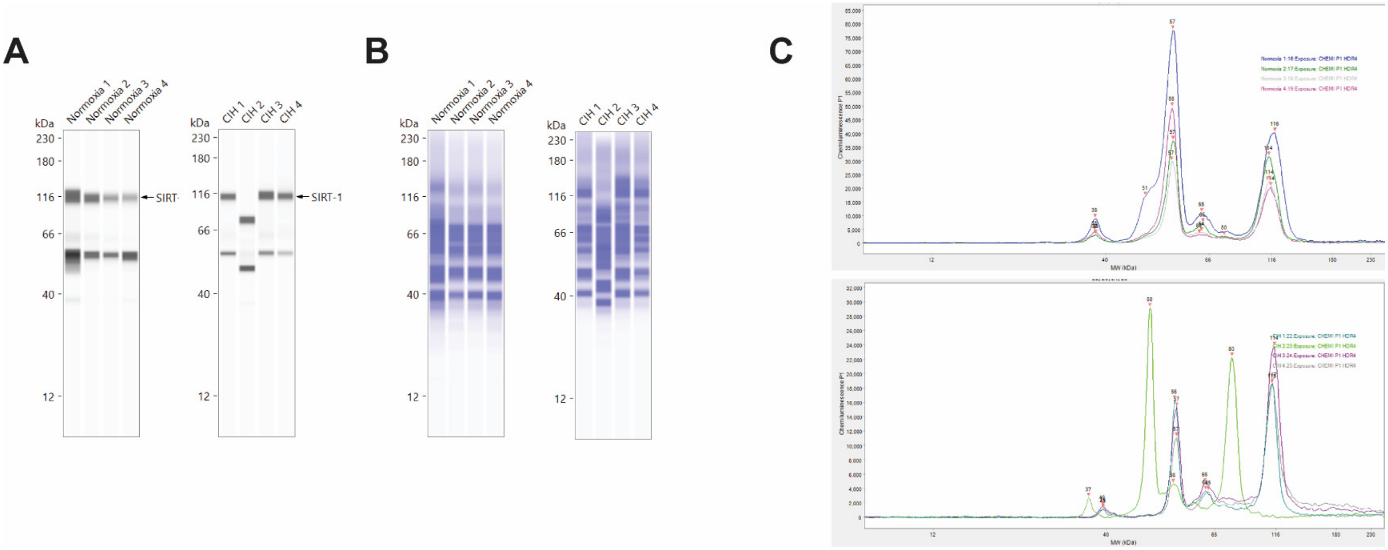

Supplementary Figure S2: Electropherograms from capillary electrophoresis assessing SIRTUIN-1 protein expression. Electropherograms from capillary electrophoresis assessing SIRTUIN-1 protein expression. A) Lane view of SIRT-1 protein expression B) Total protein per sample C) Graphical view

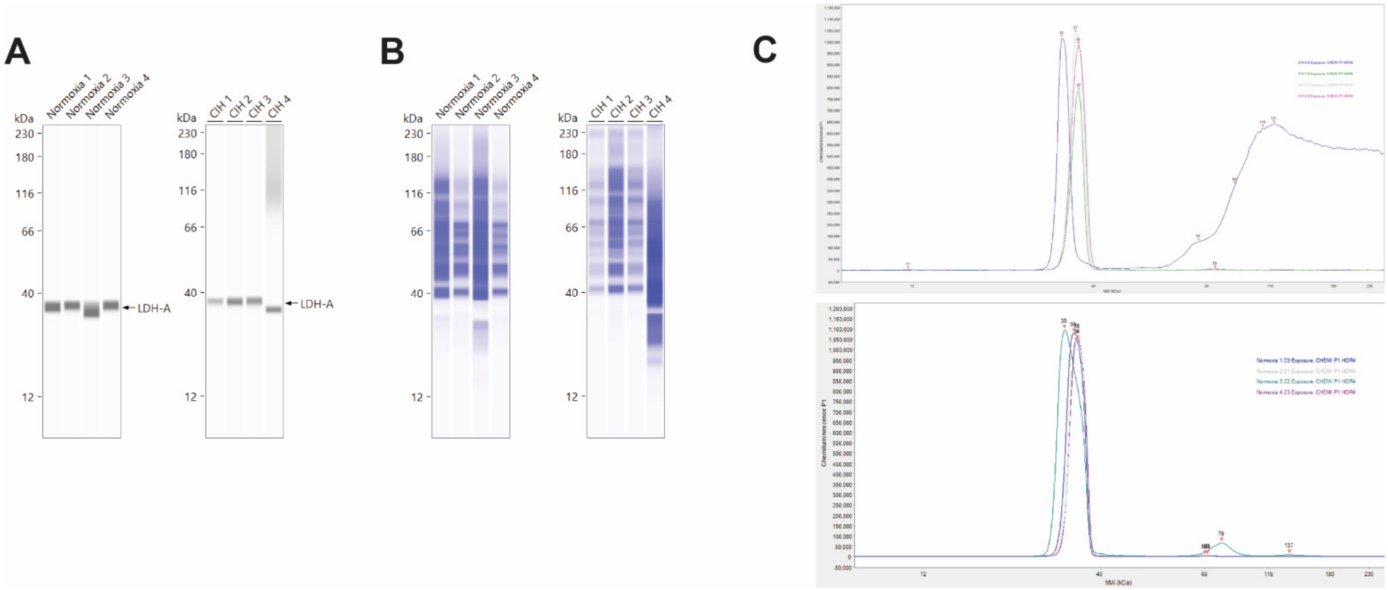

Supplementary Figure S3: Electropherograms from capillary electrophoresis assessing LDH-A protein expression. Electropherograms from capillary electrophoresis assessing LDH-A protein expression. A) Lane view of LDH-A protein expression B) Total protein per sample C) Graphical view

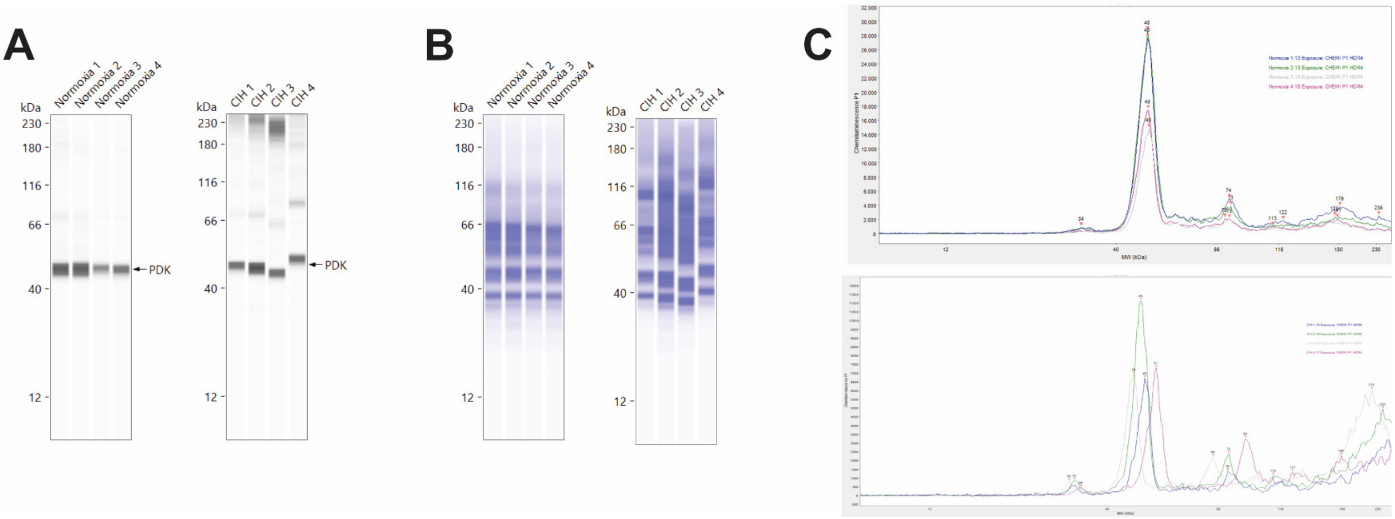

Supplementary Figure S4: Electropherograms from capillary electrophoresis assessing PDK-1 protein expression. Electropherograms from capillary electrophoresis assessing PDK-1 protein expression. A) Lane view of PDK-1 protein expression B) Total protein per sample C) Graphical view

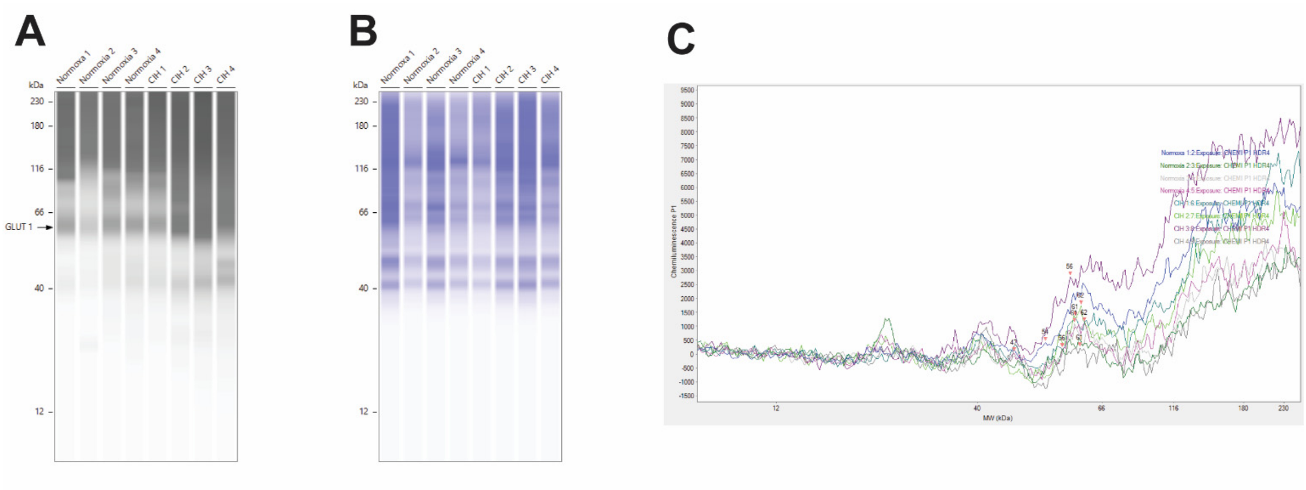

Supplementary Figure S5: Electropherograms from capillary electrophoresis assessing GLUT1 protein expression. Electropherograms from capillary electrophoresis assessing GLUT1 protein expression. A) Lane view of GLUT1 protein expression B) Total protein per sample C) Graphical view

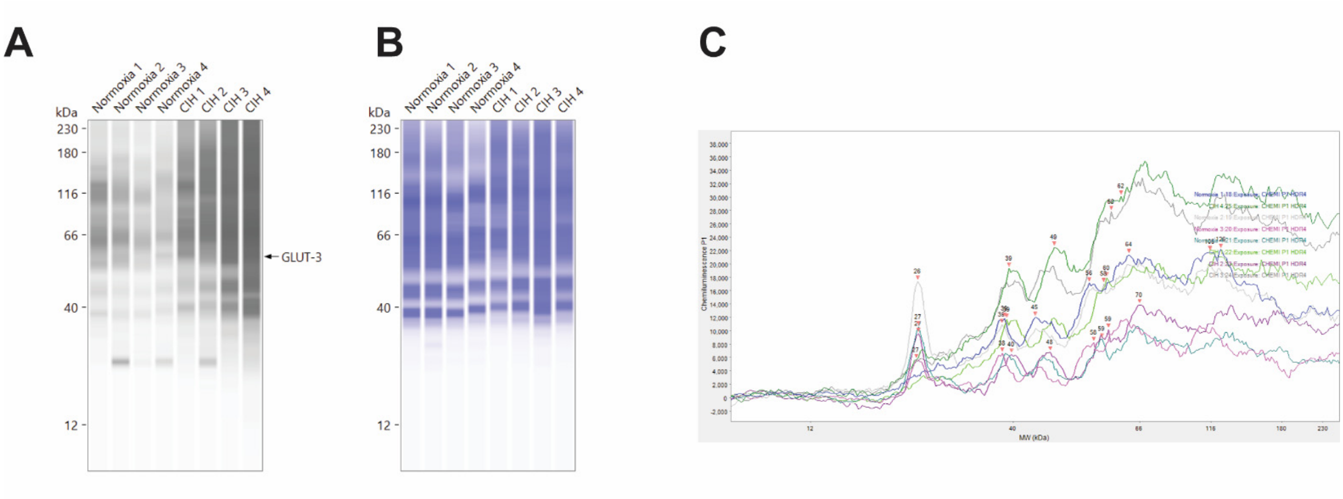

Supplementary Figure S6: Electropherograms from capillary electrophoresis assessing GLUT3 protein expression. Electropherograms from capillary electrophoresis assessing GLUT3 protein expression. A) Lane view of GLUT3 protein expression B) Total protein per sample C) Graphical view

Immunohistochemistry: Negative Controls

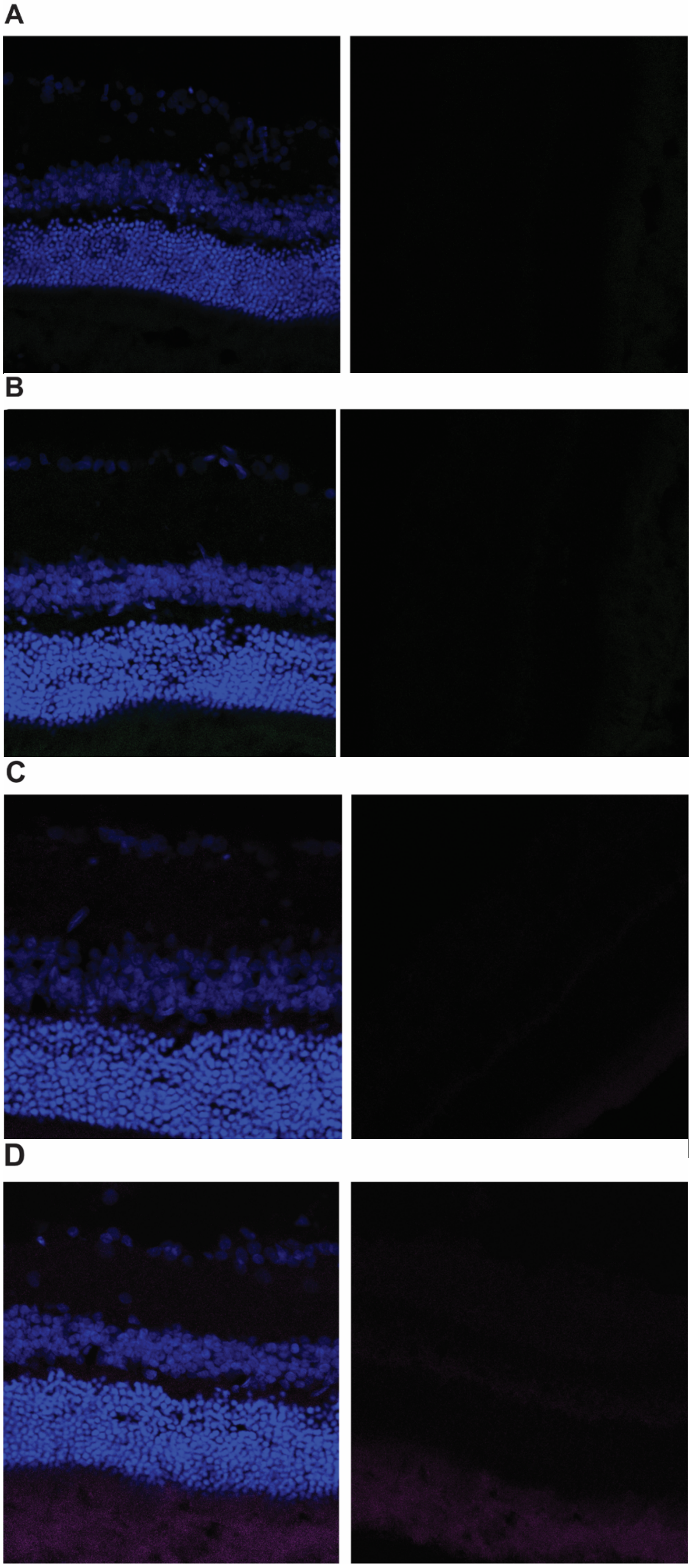

Supplementary Figure S7: No primary antibody controls for retinal sections also incubated with **A**) Anti-Mouse AlexaFluor-488, **B**) Anti-Rabbit AlexaFluor-488, **C**) Anti-Mouse AlexaFluor-647, or **D**) Anti-Rabbit AlexaFluor-647. This represents all of the secondary antibodies used. Photomicrographs on the right are also stained with DAPI.

Immunohistochemistry: Positive controls

Supplementary Figure S8: Retinal section immunolabeled with antibody against Hif-1α after four weeks of ocular hypertension.

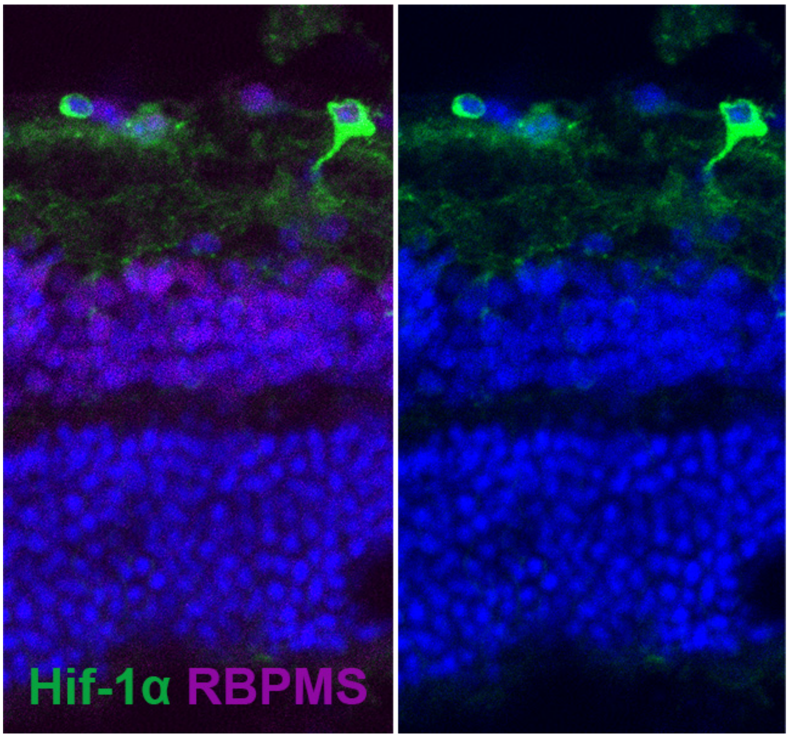

Supplementary Figure S9: Retinal section immunolabeled with antibody against 8-OHdG after four weeks of ocular hypertension.

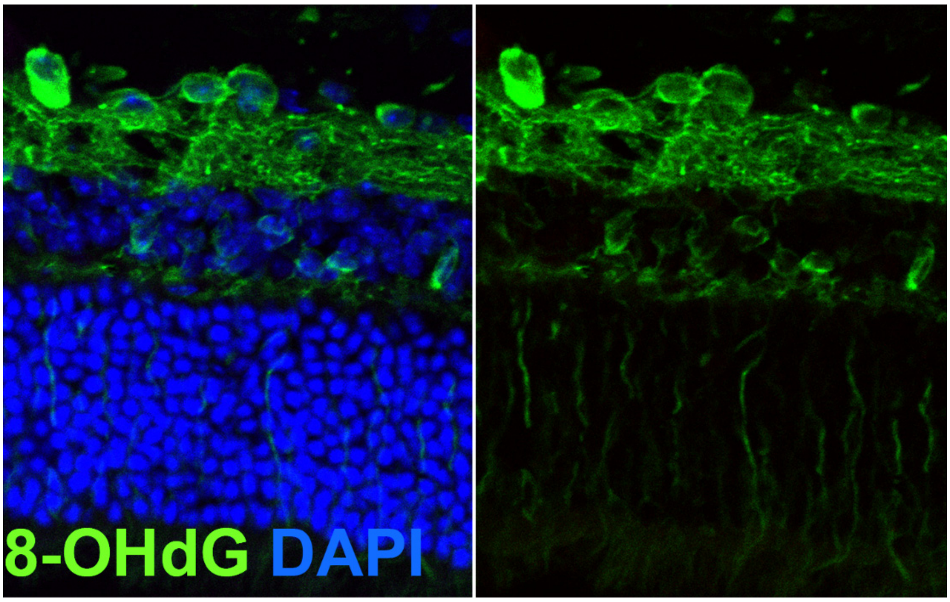

Supplementary Figure S10: Retinal section immunolabeled with antibody against TNF-α after four weeks of ocular hypertension.

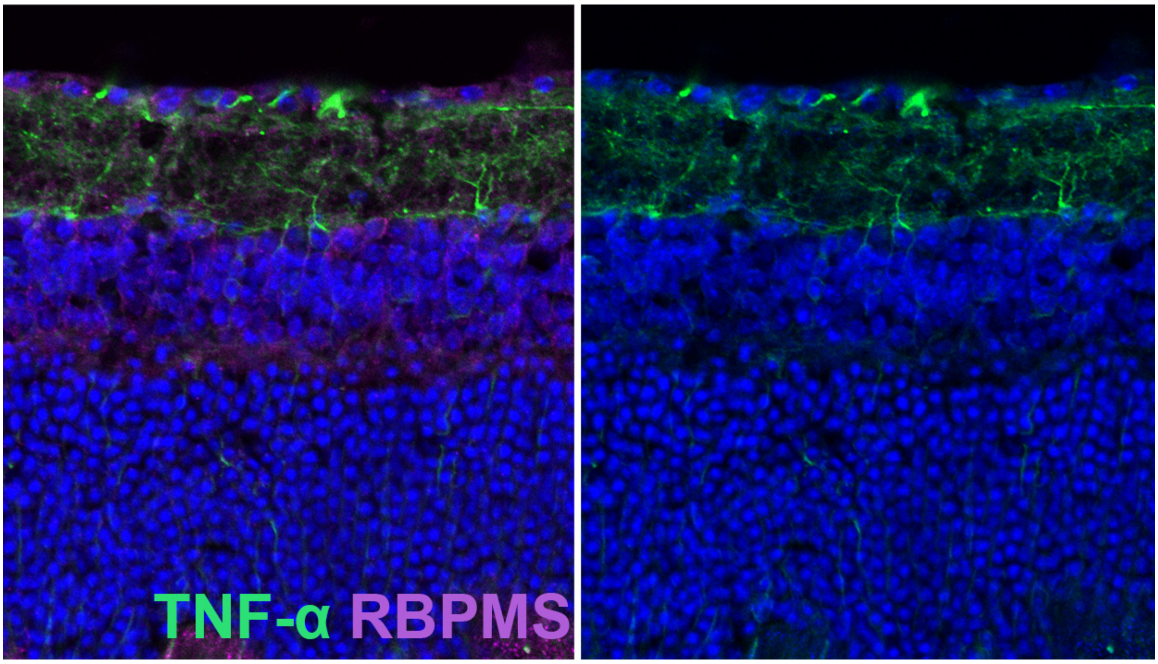

Supplementary Figure S11: Retinal section immunolabeled with antibody against Iba1 after four weeks of ocular hypertension. White arrows pointing to microglia.

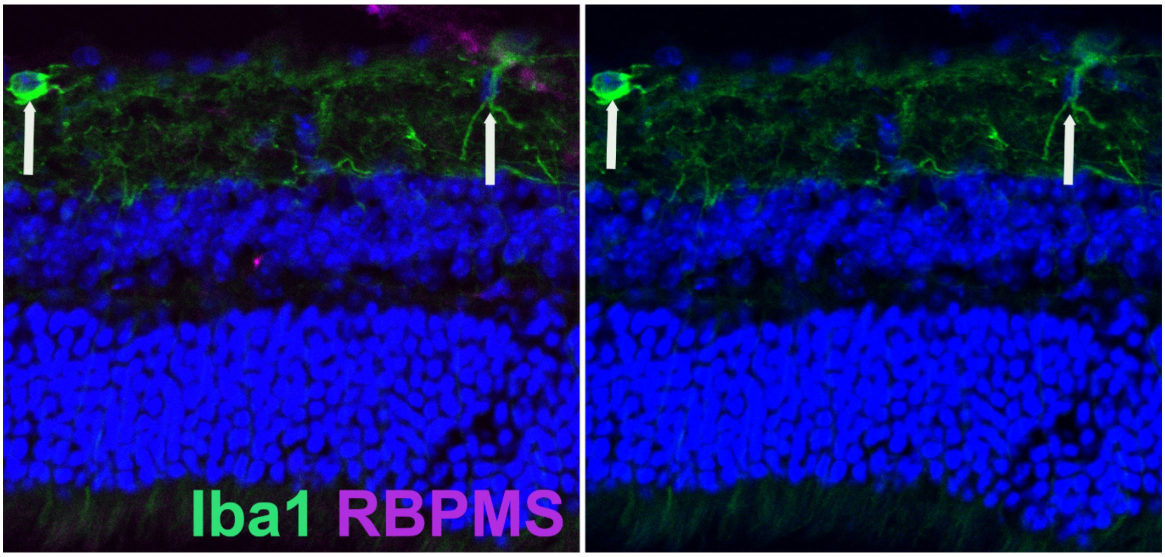

Supplement: Supplementary file 1 [file antioxidants-13-00878-s001.zip › antioxidants-3059900-supplementary.pdf]
